# Crystal Structure of ATV^*ORF*273^, a New Fold for a Thermo- and Acido-Stable Protein from the *Acidianus* Two-Tailed Virus

**DOI:** 10.1371/journal.pone.0045847

**Published:** 2012-10-08

**Authors:** Catarina Felisberto-Rodrigues, Stéphanie Blangy, Adeline Goulet, Gisle Vestergaard, Christian Cambillau, Roger A. Garrett, Miguel Ortiz-Lombardía

**Affiliations:** 1 CNRS, Aix-Marseille Université, AFMB, UMR 7257, Campus de Luminy, Marseille, France; 2 Archaea Centre, Department of Biology, University of Copenhagen, Copenhagen, Denmark; Indian Institute of Science, India

## Abstract

*Acidianus* two-tailed virus (ATV) infects crenarchaea of the genus *Acidianus* living in terrestrial thermal springs at extremely high temperatures and low pH. ATV is a member of the *Bicaudaviridae* virus family and undergoes extra-cellular development of two tails, a process that is unique in the viral world. To understand this intriguing phenomenon, we have undertaken structural studies of ATV virion proteins and here we present the crystal structure of one of these proteins, ATV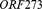. ATV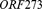 forms tetramers in solution and a molecular envelope is provided for the tetramer, computed from small-angle X-ray scattering (SAXS) data. The crystal structure has properties typical of hyperthermostable proteins, including a relatively high number of salt bridges. However, the protein also exhibits flexible loops and surface pockets. Remarkably, ATV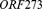 displays a new 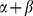 protein fold, consistent with the absence of homologues of this protein in public sequence databases.

## Introduction

Viruses are key components of biogeochemical cycles: they are the most abundant biological entities in the oceans [1] and probably on the planet. Thus, it has been estimated that every day, viruses kill about 20% of the oceanic biomass [2]. Viruses also represent a major genetic asset for the biosphere. Indeed, all organisms from each Domain of Life are likely to be infected by viruses. Although viruses infecting archaea are known since the early 1970s [3], they have only been studied in detail very recently. The notion that these viruses constitute a variety of bacteriophages with head and tail (Caudovirales), reinforced by the initial findings, was challenged by the analyses of samples isolated by Zillig and co-workers from extreme environments, rich in hyperthermophilic archaea, including the Icelandic solfatara [4]. These analyses revealed the presence of a large diversity of viral morphotypes, including viruses of linear, spindle-shaped, spherical and more exotic forms, such as drops and bottle-shapes. These viruses infect archaea living in such extreme environments, which mostly belong to the crenarchaeal orders Sulfolobales and Thermoproteales. They have been classified into eight viral families, primarily on the basis of their unusual morphotypes, subsequently backed by genomic analyses with a few viruses remaining unclassified.

Generally, crenarchaeal viruses display non-lytic life cycles, a strategy that would allow them to minimise contact to the extreme conditions of their environment. With the exception of the recently discovered *Aeropyrum* coil-shaped virus (ACV) [5], which has a single-stranded DNA genome, crenarchaeal viruses present double-stranded DNA genomes. Examination of the genomic sequences obtained so far shows that most of these viruses are unrelated to any other known viruses and that they probably have different evolutionary origins [6]. In spite of their interest from an evolutionary viewpoint, the biology of crenarchaeal viruses remains largely unexplored. This situation mirrors the fact that between 50% and 90% of the open reading frames (ORFs) predicted in the genomes of these viruses have no unambiguous functional annotations [6]. Structural analysis has been a useful tool for establishing evolutionary relationships amongst viruses [7], especially those infecting Archaea [8]. In the absence of sequence similarity to annotated proteins, structure similarity might provide insights into protein function [9]. Thus, we have worked on the structure determination of selected function–orphan crenarchaeal viral proteins with a view to obtain clues to their biological function [9]–[13].

The *Acidianus* two-tailed virus (ATV) was originally discovered in 2003 in Pozzuoli, Italy. It was isolated from a spring with temperatures higher than 85°C and at a pH of 1.5, where its host, the crenarchaeon *Acidianus convivator*, thrives [14], [15]. ATV is a member of the *Bicaudaviridae* family of crenarchaeal viruses. It has a circular double-stranded DNA genome of 62,730 base pairs, including 72 predicted open reading frames (ORFs), most without *bona fide* homologues in public sequence databases. Exceptionally for a crenarchaeal virus, ATV is known to undergo both lysogenic and lytic life cycles. Lysogeny can be interrupted and transformed into a lytic pathway by environmental stress factors. For example, ATV lytic propagation can be induced by lowering the temperature of the cultures from 85°C to 75°C [14].

Released ATV virions are initially spindle-shaped particles. At striking variance with all other known viruses, ATV undergoes an extracellular morphological transformation: between one hour and a few days, two tails develop irreversibly at each end of the particle. Tail development seems to depend solely on temperature, which must be close to that of the host's habitat (75°C to 90°C) [14]. Although infection of host cells by ATV has not yet been directly observed, it is possible that the tails facilitate or mediate the attachment of virions to host membranes.

The mechanism behind ATV's ability to grow bipolar tails has yet to be understood. At least 11 proteins have been identified in ATV virion preparations [15]. ATV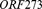 is the fourth most abundant of these proteins [15]. Notably, ATV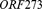 has no homologues in the distantly related virus *Sulfolobus tengchongensis* Spindle-shaped Virus 1 (STSV1), a virus that does not undergo cell–independent tail development [16]. To gain insight into this unique biological phenomenon, we have characterised the structure and the behaviour in solution of the ATV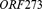 protein.

## Results and Discussion

### ATV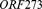 structure belongs to a new fold

ATV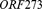 is an acidic protein (theoretical pI = 4.8) with a molar mass of 32154 Da. This protein (UniProt:Q3V4T6) was isolated from ATV virions and identified by N^′^-terminal Edman degradation from an SDS-PAGE band migrating with an apparent molecular weight of 38 kDa [15]. All the work reported here was carried out using a recombinant ATV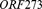 protein (molar mass 32977 Da, theoretical pI = 5.0) expressed in *E. coli* T7 Iq pLysS cells (New England Biolabs). Recombinant ATV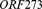 migrates on SDS polyacrylamide gels with an apparent 39 kDa molecular weight.

Two crystal forms of recombinant ATV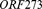 were obtained belonging to the tetragonal space group 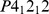 but with different cell parameters (Table 1). Crystals of the first form diffracted to 3.85 Å resolution and included one monomer in the asymmetric unit. The crystals of the second form reached a 2.15 Å diffraction limit and comprised two monomers in its asymmetric unit. Since ATV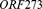 contains a single, N^′^-terminal methionine a triple mutant introducing three methionine residues was produced to facilitate SeSAD phasing. Thus, residues Leu31, Leu117 and Leu240 were selected for mutation into methionines based on their similar properties (bulky, hydrophobic) and on their predicted positioning in ordered 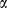-helices by PSIPRED [17]. The SeMet substituted triple mutant protein ATV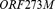 was also produced in *E. coli*, purified, and the incorporation of SeMet was checked by mass spectrometry. The SeMet protein produced crystals of the second form with cell parameters isomorphous to those of the corresponding native crystals (Table 1).

**Table 1:** Summary of data collection, phasing and refinement statistics.

	Form 1	*native* Form 2	*SeSAD* Form 2
**PDB code**	4ats	4art	
**Data collection** a			
**Beamline (synchrotron)**	ID14eh4 (ESRF)	ID29 (ESRF)	Proxima1 (Soleil)
**Wavelength (Å)**	1.1810	0.9184	0.9791
**Space group**	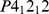	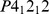	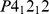
**Unit cell axes (Å)**	a = b = 101.0, c = 53.0	a = b = 78.6, c = 189.4	a = b = 78.9, c = 188.9
**Resolution (Å)**	71.43 – 3.85 (3.86 – 3.85)	50.00 – 2.15 (2.20 – 2.15)	45.00 – 2.77 (2.85 – 2.77)
**Unique reflections**	2843	32549	28786
**Multiplicity**	11.0 (11.9)	12.8 (13.4)	7.6 (7.2)
**Completeness (%)**	98.6 (100.0)	97.9 (99.9)	99.8 (98.1)
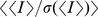	20.5 (6.8)	24.0 (3.7)	16.9 (3.1)
**R** 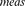 b	15.1 (65.9)	7.7 (77.4)	10.0 (74.4)
**Phasing**			
**Anom. corr. (%)** c			44 (3.74)
**Phasing power** d			1.01 (3.74)
**Refinement** a			
**Reflections used** e	2819 (459)	32529 (1660)	
**Protein atoms**	1960	3917	
**Solvent atoms**	0	281	
*R* 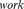 */R* 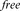 (%)^f^	27.1/29.5 (29.1/32.1)	20.2/23.4 (22.9/25.5)	
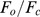 **correlation coefficient** g	0.861 (0.838)	0.947 (0.933)	
**r.m.s.d. (bonds)** h	0.009	0.010	
**r.m.s.d. (angles)** h	1.08	1.05	
**Average isotropic ** ***B*** ** factors (Å** ^2^ **)**			
**Main chain**	132.7	44.5	
**Side chains**	148.8	54.8	
**Solvent**		51.9	
**Ramachandran plot statistics (%)**			
**Residues in favoured regions**	223 (97.4)	453 (99.3)	
**Residues in allowed regions**	6 (2.6)	3 (0.7)	
**Outliers**	0 (0)	0 (0)	
**Molprobity score (percentile)** i	1.91 (100)	1.48 (98)	

a Values in parentheses are for the highest resolution shell.

b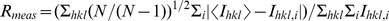 is the multiplicity (N) independent R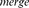.

c Mean correlation factor between two random subsets of anomalous intensity differences. In parenthesis, resolution at which anomalous correlation drops below 35%.

d Value in parenthesis: resolution for which phasing power drops below 1.0.

e In parenthesis, number of reflections randomly assigned to the test set.

f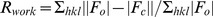. R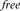 is defined as R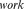 for the test set.

g The value in parentheses corresponds to the test set reflections.

h Root mean square deviation from the standard values.

i As reported by the Molprobity server [57]. In parenthesis, the percentile of this value among structures of comparable resolution.

The SeMet data extended to 2.77 Å resolution. Its structure was solved by the SAD method with data collected at the selenium anomalous peak. The data were of sufficient quality to locate the 6 Se sites present in the two molecules in the asymmetric unit. The current model is refined against native data to 2.15 Å, resulting in an R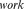/R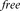 factors of 20.2%/23.4% (Table 1) and includes residues 22 to 270 in monomer A and 25 to 269 in monomer B. Two loops could not be built in the model owing to the lack of supporting electron density. The first of these loops comprises residues Ser46 to Thr53 in monomer A and Arg44 to Ile54 in monomer B, whereas the second missing loop encompases residues Gly148 to Arg151 in both monomers. The monomer A from this model was then used to solve the structure of the first crystal form by molecular replacement. Refinement against these data produced a new model, at 3.85 Å, with R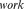 and R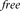 factors of 27.1% and 29.5%, respectively (Table 1). The second crystal form being solved at higher resolution, the rest of the structural description will be based on this current model, unless otherwise stated.

**Figure 1:**
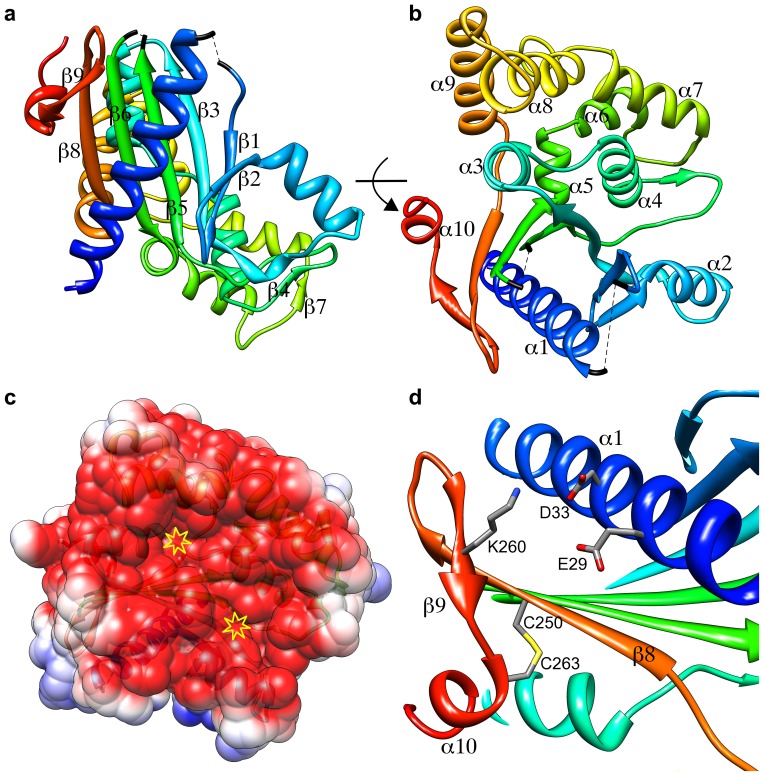
Crystal structure of ATV 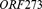
 **.** **a**) Ribbon representation of the structure of a monomer of ATV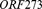. Its 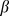-strands are labelled. The secondary structure elements are colored from the N^′^- (blue) to the C^′^-terminus (red). The loops that were not modelled are represented by dashed, black lines. **b**) A view orthogonal to the previous one, showing the arrangement of the 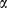-helices. This view shows part of the concave face of the monomer (top, right) **c**) The solvent-accessible surface of the ATV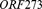 monomer concave face, colored by its electrostatic potential (red: −52 mV, blue: 52 mV), calculated at pH 7 and 150 mM NaCl with APBS [56]. Two cavities are marked with yellow open stars. **d**) A detail view of monomer B showing the salt bridge network formed by residues Glu29/Asp33/Lys260 as well as the disulphide bond established by cysteine residues 250 and 263. Amino acid residues are labelled in one-letter code; the secondary structure elements to which they belong are also labelled.

The structure of an ATV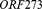 monomer is made up of 10 helices and two 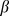-sheets, one composed of seven strands and the other consisting of two short parallel strands (Figure 1). The major 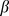-sheet is mainly antiparallel, but strands 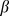
3 and 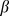
5 are parallel (Figure 1a). This 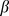-sheet forms a half-barrel with the first 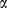-helix (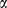
1) packed onto its concave side. The rest of the helices and the small 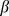-sheet are arranged at the other side of the major 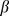-sheet (Figure 1b). The monomer can be described as a disk with overall dimensions 59 Å
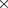
58 Å
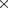
38 Å. The protein N^′^-terminus protrudes on one side of the disk, whereas the surface of the other side, lined by a high number of acidic residues (Figure 1c), is concave. The two unmodelled loops face each other at the periphery of the disk, with the visible extremes pointing towards the concave side (Figure 1a). This concave face (Figure 1c) includes two deep pockets with volumes of 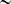
575 Å^3^ and 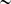
400 Å^3^, respectively, as calculated by the Relibase+ [18] implementation of LIGSITE [19]. Other smaller pockets are distributed across the protein surface. Analysis of these cavities with the SUMO server [20] did not suggest any clear-cut ligands matching them.

As expected for a hyperthermostable protein [21], the ATV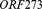 monomer is stabilised by a relatively high number of salt bridges (5 in monomer A and 8 in monomer B; to be considered as engaged in a salt bridge, the centroids of the side-chain charged groups and at least a pair of side-chain nitrogen and oxygen atoms of the ion-pairing residues must be within a 4 Å distance [22]). Some of these salt bridges form networks, namely the triplets Glu29/Asp33/Lys260 (Figure 1d) and Glu252/Lys254/Lys265 in monomer B, another feature related to hyperthermostability [21]. Furthermore, a disulphide bond is observed between cysteines 250 and 263 in both monomers (Figure 1d).

Structural similarity searches performed with Dali [23] and PDBeFold [24] produced no significant hits. The highest Z-scores were below 5 for Dali and below 4 for PDBeFold, corresponding to high r.m.s.d. values (worse than 2.7 Å) over a small number of residues ranging from 60 to 120 amino-acids. All these hits were essentially overlapping with the major 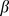-sheet and some included the 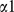 helix that is packed against this sheet. Thus, we conclude that the ATV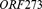 structure defines a new 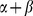 fold.

### ATV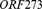 can form different types of oligomers

**Figure 2:**
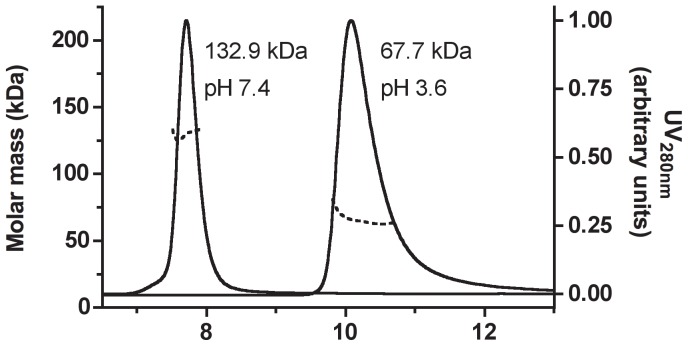
Oligomeric state of ATV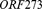 in solution. About 120 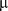
g of purified ATV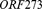 were subjected to size-exclusion chromatography coupled to MALS/RI/UV detectors as described in the Experimental section. Two chromatograms/mass analyses are combined in this figure, showing the elution of the tetrameric (left) and dimeric (right) forms of ATV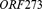, obtained at pH 7.4 and 3.6, respectively. The molar mass (dotted lines), derived from refractive index measurements, and the absorption at 280 nm (full line) were plotted as functions of the elution volume around the peaks. The weight-averaged molar mass (M*_w_*) values determined by the ASTRA software are indicated.

The elution of the recombinant ATV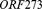 protein from the size-exclusion chromatography column used for its purification was compatible with the protein forming trimers or tetramers at pH 8.5. To define more accurately the stoichiometry of this oligomer, we analysed the purified protein by the MALS/SEC method (Figure 2). With the protein injected at 4 mg/mL (121 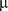
M), these experiments showed that ATV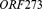 forms a tetramer (132.9
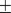
1.3 kDa) at pH 7.4 but a dimer (67.7
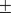
3.4 kDa) at pH 3.6. Hydrodynamic radius calculation performed with the ASTRA software yielded values of 4.84
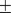
0.27 nm and 3.54
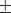
0.30 nm, respectively.

The analysis of the crystal interfaces present in the second crystal form with the PISA software [25] suggested two possible dimers with a significant buried area at the interface, namely 1258 Å^2^ and 1087 Å^2^ per monomer, respectively. Remarkably, these interfaces are not conserved in the first crystal form, which actually displays less extensive contact surfaces, the largest masking 608 Å^2^ per monomer, none of them considered as significant by the PISA algorithm.

**Figure 3:**
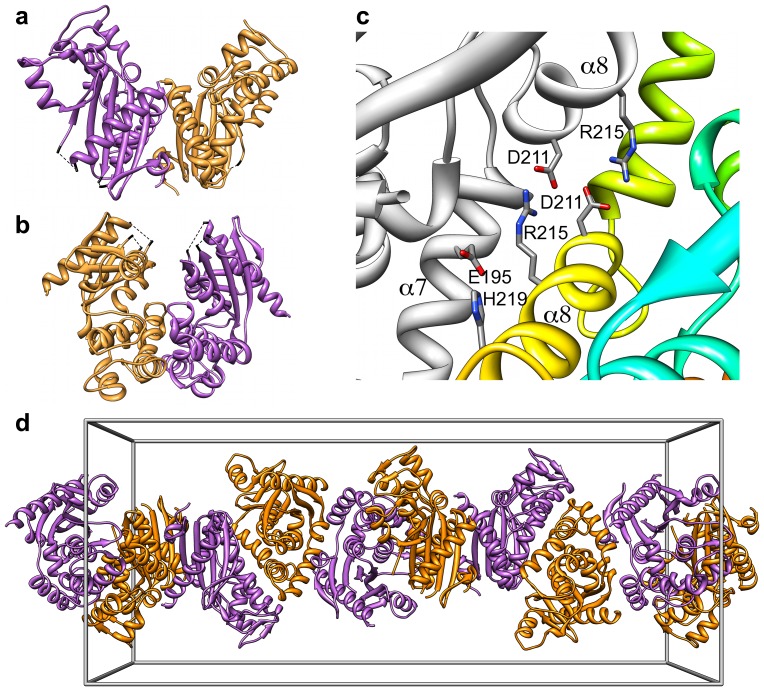
Crystal packing of ATV 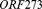
 **.** **a**) Overall view of the open and **b**) closed dimers found in the second crystal form. In both cases the pseudo-two-fold axis is vertical in the plane of the paper. **c**) View of the interface between monomer A (colored as in in Figure 1) and monomer B (grey) in the closed dimer. Amino acid residues involved in cross-monomer salt bridges are labelled in one-letter code. The secondary structure elements that support them are also labelled. **d**) A view of the dimers arranged as a continuous helical fiber in the crystal. The edges of a unit cell box are shown in grey color.

Each of the two interfaces suggested by PISA results in a possible dimer with pseudo-two-fold symmetry. Based on their appearence, we call them the ‘open’ and the ‘closed’ dimers, respectively (Figure 3a and 3b). In the closed dimer the concave sides of the monomers face each other thereby creating a chamber with a volume of 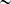
8650 Å^3^. In the open dimer the interface involves their convex side. Not only do the open and closed dimers bear similar contact surface areas, but they also display the same number (15) of inter-subunit hydrogen bonds, defined according to Mills & Dean criteria [26]. There are however two global differences between these two dimers. First, the closed dimer exhibits two strong symmetrical salt bridges between Asp211 on one monomer and Arg215 on the other. Furthermore, a third salt bridge is observed between Glu195 in monomer B and His219 in monomer A (Figure 3c). Conversely, only weak ion-pair interactions are found in the open dimer, with the best N-O bridge [22] established between residues Glu29 and His237. Second, shape complementarity of the interface surfaces, as calculated by the SC program [27] is significantly better (0.670) for the closed dimer than for the open dimer (0.563).

**Figure 4:**
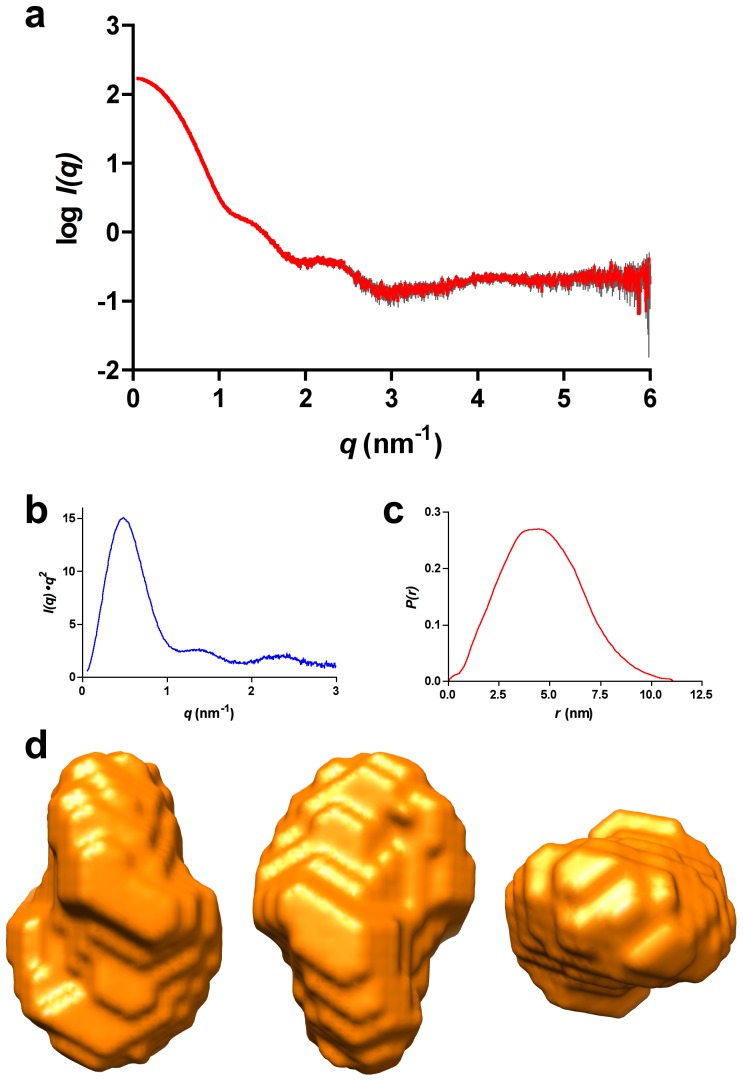
SAXS analysis of ATV 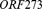
 **.** **a**) SAXS intensity as a function of the momentum transfer. This profile corresponds to the measurements taken at 4.7 mg/ml protein cocentration and pH 8.5. Average values are in red and the standard error in grey. **b**) The Kratky plot (see text for details) corresponds to a folded protein. **c**) Pair-distance distribution, *P(r)*, function of the data shown in panel a). **d**) Three orthogonal views of the *ab initio* envelope calculated imposing orthorhombic symmetry.

Noteworthy is that the dimers observed in the ATV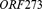 crystals do not combine to form a tetramer but rather generate fibers (see below). This observation is at odds with the fact that these crystals appear under pH conditions (pH 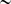
6) closer to those used for the protein purification, where the MALS/SEC data indicate the presence of tetramers, than to pH 3.6, where dimers are detected by this technique. We sought to resolve this apparent discrepancy by using SAXS to characterize the ATV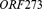 oligomer in solution and examine its shape and dimensions. Attempts to obtain SAXS data at pH 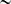
6 were unsuccessful, due to concentration-dependent protein aggregation. Therefore we collected SAXS data under the pH and ionic strength conditions used for the protein purification. Guinier analysis yielded an R*_g_* of 35.8 Å, which is bigger than values calculated for the closed (22.9 Å) and the open (24.4 Å) dimers. The maximum dimension of the particle (D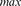 = 111.0 Å), obtained from the distance distribution function (*P(r)*), was also larger than those computed from the structures of the closed (67.9 Å) and open (84.0 Å) dimers. Finally, the excluded volume of the hydrated particle (Porod volume) was 221.5 nm^3^ that, according to the empirical formula 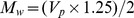 [28], gives a molar mass of 133 kDa, in good agreement with an ATV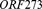 tetramer (132 kDa). The Kratky plot was consistent with a properly folded protein (Figure 4b), whereas the *P(r)* function was monomodal and suggested a compact, slightly elongated particle (Figure 4c).

Next we employed the program DAMMIF to carry out *ab initio* shape reconstruction of the oligomer. We conducted several series of independent runs with either no forced symmetry or imposing P2, P222, P3 or P4 symmetries (Table 2, Figure S1a). Within each symmetry class, the models were very reproducible with average normalized spatial discrepancy (NSD) values below 1.0, consistent with structurally similar solutions. Furthermore, all the models were similar in terms of agreement with the experimental data, as measured by DAMMIF 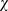 parameter. Slightly better agreement was attained with P1 and P222 (Figure 4d) models, whereas P4 models fitted the data systematically worse than the rest. We note that the averaged models have slightly bigger volumes, ranging from 256.5 Å (P4 model) to 282.5 Å (P1 model), than that obtained by Porod analysis.

**Table 2:** Summary of SAXS results.

DAMMIF^a^					
Symmetry	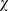	NSD	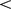 Vol 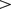 (nm^3^)	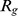 (Å)	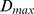 (Å)
**P1**	1.654 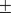 0.011	0.583 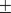 0.019	282.5	34.4	115
**P2**	1.659 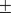 0.021	0.680 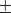 0.106	278.0	34.2	115
**P222**	1.648 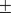 0.008	0.486 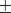 0.017	272.2	34.7	114
**P3**	1.704 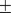 0.036	0.654 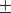 0.032	278.4	33.6	102
**P4**	1.770 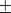 0.052	0.768 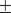 0.127	256.5	32.8	90

a *ab initio* shape reconstruction.

b Rigid-body modelling.

In a complementary approach, we used SASREF [29] to generate rigid-body refined models based on the available structures of ATV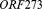, that is the monomer and the two possible dimers identified in the second crystal form. For the monomer we used the same symmetries as for the *ab initio* shape reconstruction, whereas for the dimers we calculated models with P1 and P2 symmetry. As expected, better agreement to the experimental curve was achieved by the trials with more degrees of freedom, i.e. those involving the monomer (Table 2), with the notable exception of the P3 symmetry, which gave the worst agreement of all the rigid-body models. Examination of the P3 models showed that the monomers are disconnected since they need to occupy a volume that is better accounted for by four monomers. Amongst the models generated from the monomers, those with P4 symmetry performed only better than the P3 models and worse than the rest, in terms of 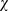 (Table 2). To further explore the possibility that the protein may form trimers in solution and to exclude a problem with SASREF for the generation of appropriated trigonal models, we used the symmdock software [30] to generate trimers of ATV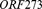 with trigonal symmetry. The best symmdock model has a score of 11592 compared to a score of 8892 for the second best model, with scores monotonously decreasing thereafter. The best symmdock model has an R*_g_* of 25.8 Å, the second best model has R*_g_* = 25.9 Å and the average R*_g_* of the best 10 models is 27.3
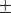
1.0 Å. Similarly, the first model has D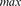 = 84.5 Å, the second model has D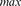 = 83.8 Å and the average of the best 10 models gives D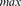 = 84.3
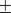
2.8 Å. These values are clearly different from the experimental values obtained by SAXS analysis. Further, the SAXS profiles calculated from these trimers by the program CRYSOL [31], fitted very poorly the experimental profile, with average 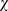 = 39.98
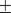
6.73. We conclude that under these experimental conditions ATV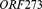 does not form trimers.

Interestingly, the rigid-body refined models calculated from the crystallographic open dimer came next to those from the monomer in terms of agreement with the SAXS data. Furthermore, models calculated from the open dimer were all very similar, irrespective of the symmetry used (overall 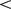
NSD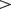 = 1.128
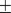
0.492). We conclude from these results that under the conditions tested (pH 8.5, 100 mM NaCl) ATV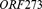 forms a tetramer, possibly with point group 222 symmetry (Figure S1b).

### ATV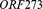 is a hyperthermostable and hyperacidostable protein

**Figure 5:**
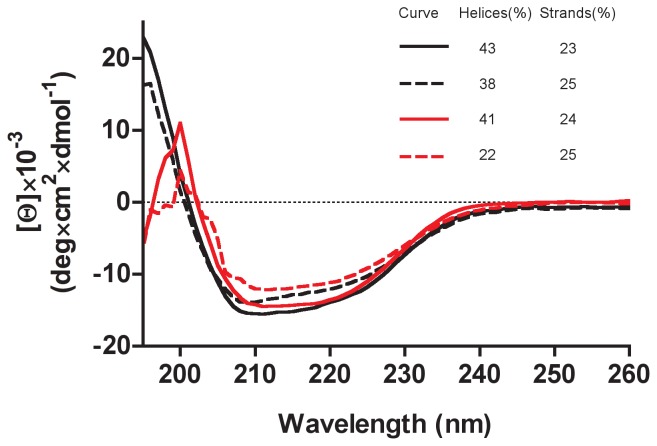
Circular dichroism spectra of ATV 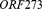
 **.** Mean residue ellipticity spectra recorded at pH 7.2 (black lines) and pH 0 (red lines), either at 20°C (full lines) or at 80°C (dashed lines). The content in helices and strands, as determined by deconvolution of the spectra, is shown (see text for details).

ATV viruses proliferate at extreme pH (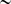
1.5) and temperature (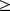
85°C). Under these conditions, structural proteins have to bear an extreme stability to preserve fold and function. The ATV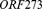 CD spectrum recorded at 20°C and pH 7.2 displays two minima at 215 and 222 nm, characteristic of a folded protein containing mainly 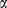-helices (Figure 5). The CD spectra recorded at 20°C and 80°C, both at neutral and acidic (pH 0) conditions are all nearly superimposable, establishing the extreme protein resistance to high temperature, low pH and a combination of both factors. Deconvolution of the CD spectra using the CDSSTR program [32], as implemented in the Dichroweb server [33], was consistent with the secondary structure derived from the crystal structure (42% helices and 18% strands, see Figure 5) within the uncertainties of this approach [32]. These analyses suggest that, at the two pH values studied, increasing the temperature to 80°C slightly destabilises the protein helices without affecting its content in 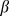-strands.

In contrast, at pH 11 and 80°C, ATV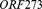 unfolds: the signal at 200 nm is negative and the signal in the range 215–260 nm is closer to zero (not shown). Importantly, the fact that ATV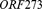 withstands a combination of low pH and high temperature agrees well with the environment of the ATV virus in the thermal spring where it was discovered.

### Final remarks

Apart from an increased presence of salt-bridges and other ionic pair interactions, ATV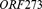 bears some rare traits for a hyperthermostable protein (see [21] for a thorough discussion of these properties). First, its fold is less compact than expected and displays several cavities. Second, its C^′^-terminus and especially its N^′^-terminus are not well structured. Finally, it carries disordered loops. The presence of cavities and of disordered loops would seem to suggest an enzymatic function. However, the novelty of the fold and the fact that the protein sequence does not retrieve any significant hit in public databases hinder the assignment of a biological function to ATV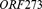. Furthermore, although the protein has a number of surface pockets we could not assign them to any *bona fide* ligand/substrate. Significantly, ATV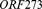 is the fourth most abundant protein in virion preparations [15] and, moreover, no homologue of ATV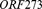 is present in the STSV1 virus [16]. In contrast, the ATV homologue (ATV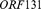) of the STSV1 major capsid protein (STSV1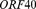) is also the most abundant protein in the ATV virion [15].

The two viruses ATV and STSV1 both exhibit large fusiform bodies but while the former generates long bipolar tails extracellularly, the latter generates one long tail intracellularly [14], [16]. Both genomes encode several pairs of homologous proteins, albeit distantly related, but they differ markedly in their virion protein contents. Whereas ATV virions contain several major protein components, STSV1 carries only one major component, the coat protein STSV1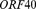. It has been hypothesised that some of the virion components of ATV actively contribute to the extracellular tail development [15] and in a detailed study of one of the major components, a MoxR-type ATPase ATV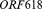, evidence was provided for a co-chaperone activity together with a Von Willebrand domain A protein ATV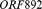 [34]. Moreover, a model was presented whereby this co-chaperone facilitated tail development together with another virion protein ATV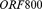, which exhibits intermediate filament-like properties. Further, it was proposed that novel ATV DNA binding proteins, also present in the virions, were involved in drawing DNA along the tails [34]. In this context, a structural role for ATV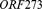 cannot be ruled out in spite of its enzyme-like features. In this regard, the stability of ATV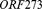 to extreme acidic pH suggests that it may be in contact with the external environment of the virion and thus participate in its coat and contribute to the development of ATV tails. Finally, the two interfaces identified in the second-form crystals of ATV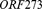 are assembled head-to-tail, resulting in helicoidal fibers that extend indefinitely along the crystallographic *c*-axis (Figure 3d). Although we have not observed fiber formation when handling the protein, such a process could be dependent on pH. In this respect, the crystal pH (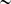
6) might reflect the protein natural environment better than the pH (8.5) of the protein solutions. Our results should help guiding further experiments necessary to understand the biological function of ATV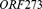 and its possible involvement in the development of ATV tails.

## Materials and Methods

### ATV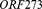 construction and purification procedures

The ATV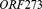 gene was cloned into the pDEST14 expression vector according to standard Gateway protocols. The final construct included a coding sequence for a C–terminal hexa–histidine tag. A variant of ATV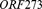 (ATV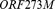), carrying the Leu31Met, Leu117Met and Leu240Met mutations, was generated by syntetic production of the mutated gene (Geneart).

Plasmids were transformed into the *Escherichia coli* T7 Iq pLysS expression strain (New England Biolabs). Cells were grown at 37°C in Lysogeny Broth (LB) until the OD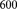 reached 0.6. Protein expression was induced then with 0.5 mM isopropyl-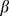-thio-galactoside (IPTG) and the cultures maintained at 25°C. After 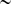
16 hours, cells were harvested and lysed by sonication in 50 mM sodium phosphate buffer (pH 8), 300 mM NaCl, 10 mM imidazole and a protease inhibitor cocktail (Complete EDTA-free, Roche). Soluble protein was separated from inclusion bodies and cell debris by centrifuging for 30 min at 20,000 *g*. We used an ÄKTA FPLC system for a two-step purification. First, the lysates were applied onto a Ni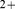 affinity chromatography column (HisTrap 5 ml, GE Healthcare) and eluted with 250 mM imidazole in 50 mM sodium phosphate buffer (pH 8) and 300 mM NaCl. A preparative Superdex 200 (GE Healthcare) gel filtration column was then run in 10 mM Bicine pH 8.5, 100 mM NaCl to remove aggregated material.

Seleno-Methionine-labeled ATV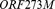 was prepared following standard procedures in the minimum medium M9 by blocking the methionine biosynthesis pathway [35]. Expression, purification, and characterisation of the SeMet-labeled ATV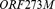 protein were carried out using the same protocols as for the native protein.

### Size-Exclusion Chromatography-coupled Multi-Angle Light Scattering

Size-Exclusion chromatography (SEC) was performed on an Alliance 2695 HPLC system (Waters). A Shodex KW803 column, operated at 0.5 mL/min, was used in either 20 mM Hepes (pH 7.4) with 100 mM NaCl or in 20 mM citrate buffer (pH 3.6) with 150 mM NaCl. Multi-Angle Light Scattering (MALS), Ultra-Violet (UV) spectrophotometry, Quasi-Elastic Light Scattering (QELS) and Refractive index (RI) measurements were achieved with MiniDawn Treos (Wyatt Technology), Photo Diode Array 2996 (Waters), DynaPro (Wyatt Technology) and Optilab rEX (Wyatt Technology) detectors, respectively. Weight-averaged molar mass (M*_w_*) and hydrodynamic radius calculations were performed with the ASTRA software (Wyatt Technology) using a dn/dc value of 0.185 mL/g.

### Circular Dichroism

Temperature and pH stability studies were carried out by far-UV Circular Dichroism (CD) spectroscopy. CD spectra were recorded with a JASCO J-810 spectropolarimeter (JASCO Corporation, Japan) equipped with a Peltier temperature control system. Far–UV measurements (195–260 nm) were performed using a 0.1 cm path quartz cuvette, with a scanning speed of 20 nm/min, spectral bandwidth of 1 nm, and were averaged over three scans. The solvent spectra were subtracted in all experiments to eliminate background effects. CD measurements in millidegrees were performed at a protein concentration of 0.15 mg/mL in 10 mM sodium phosphate buffer at pH 7.2. Stability tests at pH 0 and pH 11 were carried out in 1 M HCl and 1 mM NaOH, respectively. Thermal denaturation was monitored by increasing the temperature from 20°C to 80°C.

The CD spectra were deconvoluted by using the CDSSTR program [32], with reference database 7, as implemented in the Dichroweb server [33]. The normalised root-mean square deviations were in the 0.004–0.013 range, consistent with excellent agreement between the experimental data and the fitting from the deconvolution.

### Small-Angle X-ray Scattering measurements and analysis

All Small-Angle X-ray Scattering (SAXS) measurements were carried out at the ID14eh13 beamline (ESRF, Grenoble, France) at a working energy of 13.32 keV corresponding to 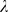 = 0.931 Å. Data were collected on a Pilatus 1 M detector placed at a sample-detector distance of 2.43 m.

SAXS data were collected using 30 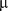
l of protein solution at 2.3, 4.7 and 9.2 mg/ml in 10 mM Bicine (pH 8.5) buffer with 100 mM NaCl, loaded by a robotic system into a 2-mm quartz capillary mounted in a vacuum. This procedure enables the sample to move across the beam during exposure thus minimizing the effect of radiation damage. Ten exposures each of 10 s were made in this way for each condition. Individual frames were processed automatically and independently at the beamline by the data collection software (BsxCUBE), yielding radially averaged normalized intensities as a function of the momentum transfer q, with 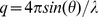, where 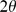 is the total scattering angle and 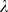 is the X-ray wavelength. Data were collected in the range q = 0.04–6 nm^−1^. The ten frames were combined to give the average scattering curve for each measurement and any data points affected by aggregation, possibly induced by radiation damage, were excluded. Scattering from the buffer alone was also measured before and after each sample measurement and the average of these two buffer measurements was used for background subtraction using the program PRIMUS [36] from the ATSAS package [37]. PRIMUS was also used to perform Guinier analysis [38] of the low q data, which provides an estimate of the radius of gyration (R*_g_*). Regularized indirect transforms of the scattering data were carried out with the program GNOM [39] to obtain *P(r)* functions of interatomic distances. The *P(r)* function has a maximum at the most probable intermolecular distance and goes to zero at D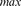, the maximum intramolecular distance. Values of D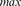 were chosen that yielded solutions that fit the experimental data well and have a smooth and strictly positive *P(r)* function. This approach also allows the calculation of R*_g_* values that agreed with the values found by the Guinier analysis.

### *Ab initio* 3D shape reconstructions

We built 3D bead models fitting the scattering data with the program DAMMIF [40]. Ten independent DAMMIF runs were performed for each scattering profile, with data extending up to 0.25 Å^−1^, using slow mode settings, assuming either P1, P2, P222, P3 or P4 symmetry and allowing for a maximum 500 steps to grant convergence. The models resulting from independent runs at each symmetry were superimposed using the DAMAVER suite [41]. This yielded an initial alignment of structures based on their axes of inertia followed by minimisation of the normalized spatial discrepancy (NSD), which is zero for identical objects and larger than 1 for systematically different objects [42]. The aligned structures were then averaged, giving an effective occupancy to each voxel in the model, and filtered at half-maximal occupancy to produce models of the appropriate volume that were used for all subsequent analyses. To provide a clearer representation of the 3D shape reconstructions, bead models were converted to density maps by the program pdb2vol from the Situs package [43].

### Rigid body modelling of the SAXS data

We used the program CRYSOL [31] to generate theoretical scattering curves from monomer A of the best resolution model of ATV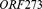, as well as from the two putative dimers observed in this crystal form. Rigid body modeling was performed with the program SASREF [29], which uses a simulated annealing protocol to build an interconnected ensemble of subunits without steric clashes, while minimizing the discrepancy between the experimental scattering data and the curves calculated from the appropriate subunits by CRYSOL.

### Crystallisation and Structure Determination

ATV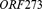 crystallisation trials were carried out in sitting-drop vapour diffusion method at 20°C in 96-well Greiner crystallisation plates using a nanodrop-dispensing robot (Cartesian Inc.). The first crystals, belonging to space group 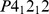 (Table 1), were obtained in 5%–15% PEG 8000, 0.2 M MgCl_2_, 0.1 M Tris (pH 7–8). A native data set (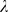 = 0.91839 Å) to 3.85 Å resolution was collected at the ID14eh4 beamline (ESRF, Grenoble, France).

Crystals of a second form grew in a few days by mixing 1.5 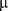
L protein at 5 mg/mL with 0.5 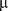
L 3.6% isopropanol, 1.9 M (NH_4_)_2_SO_4_, 5 mM MgCl_2_, 2 mM AMP. The mother liquor was pH 6. Crystals were cryoprotected with mother-liquor supplemented with 25% glycerol and 2.3 M (NH_4_)_2_SO_4_ and flash vitrified in liquid nitrogen. Two data sets were collected: a native data set (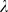 = 0.91839 Å) to 2.15 Å resolution at the ID29 beamline (ESRF, Grenoble, France) and a Se-SAD data set (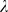 = 0.97911 Å) to 2.77 Å at the Proxima 1 beamline (SOLEIL, Gif-sur-Yvette, France).

Data integration and scaling were done using the XDS package [44] and POINTLESS [45] was used to help establishing the space group. The structure of ATV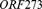 was solved by the single-wavelength anomalous diffraction (SAD) method using the autoSHARP program [46] with SHELXD [47] to locate the selenium substructure. Initial automatic building was performed with Buccaneer [48]. Alternative cycles of manual model building with Coot [49] and refinement with either autoBuster-TNT [50] or refmac5 [51] were carried out to improve the initial model.

We solved the structure of the first crystal form by molecular replacement with the program PHASER [52], using as template the final model from the second crystal form. Refinement was performed with autoBuster-TNT [50] with the “target” option [53] that uses local similarity restraints to a separate already determined structure, typically at higher resolution, that remains fixed during the refinement of the structure being refined. This procedure facilitates the refinement of low resolution structures. Our final model from the second crystal form was used as “target” structure. Temperature factors were refined with the translation-libration-screw (TLS) approach with a single TLS group.

We used the DSSP program [54] to define the secondary structure elements of the higher resolution crystal structure. Figures were generated using Chimera [55].

## Supporting Information

Figure S1
**SAXS analysis of ATV** 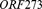 **.** **a**) Three orthogonal views of each of the five *ab initio* envelopes calculated imposing (from left to right) no, binary, orthorhombic, trigonal or tetragonal symmetry. **b**) Fitting of the SASREF model obtained from the open dimer using P2 symmetry into the P222 DAMMIF model. The fitting was performed by the program Chimera [55].(TIF)
