# Supplementary figures and images for: Crystal Structure of ATVORF273, a New Fold for a Thermo- and Acido-Stable Protein from the Acidianus Two-Tailed Virus
Source: PLoS One. 2012 Oct 8;7(10):e45847. doi: 10.1371/journal.pone.0045847 (PMC3466262; doi:10.1371/journal.pone.0045847)

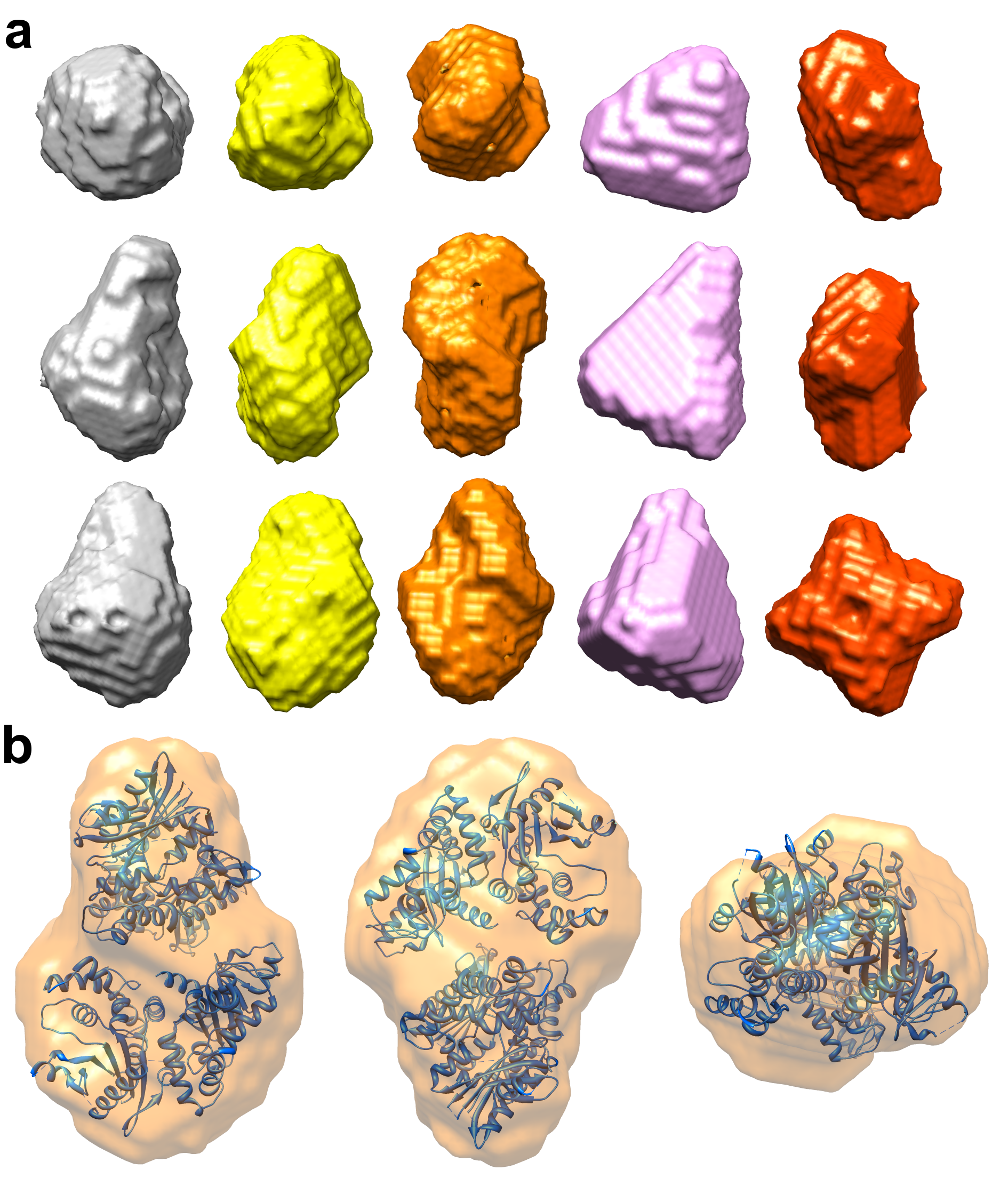

Supplement: Figure S1 — SAXS analysis of ATV . a) Three orthogonal views of each of the five ab initio envelopes calculated imposing (from left to right) no, binary, orthorhombic, trigonal or tetragonal symmetry. b) Fitting of the SASREF model obtained from the open dimer using P2 symmetry into the P222 DAMMIF model. The fitting was performed by the program Chimera [55]. (TIF) [file pone.0045847.s001.tif]
